# Phylogeography of *Populus koreana* reveals an unexpected glacial refugium in Northeast Asia

**DOI:** 10.48130/FR-2023-0023

**Published:** 2023-09-27

**Authors:** Ji Wang, Hongying Zhang, Markus Ruhsam, Xiaoyan Fan, Xue Li, Jae Min Chung, Mi Yoon Chung, Myong Gi Chung, Shiyang Wang, Jing Wang, Kangshan Mao

**Affiliations:** 1 Key Laboratory of Bio-Resource and Eco-Environment of Ministry of Education and State Key Lab of Hydraulics and Mountain River Engineering, College of Life Sciences, Sichuan University, Chengdu 610065, China; 2 Royal Botanic Garden Edinburgh, 20A Inverleith Row, Edinburgh EH3 5LR, UK; 3 Department of Garden and Plant Resources, Korea National Arboretum, Pocheon 11186, Republic of Korea; 4 Department of Biological Sciences, Chungnam National University, Daejeon 34134, Republic of Korea; 5 Division of Life Science and the Research Institute of Natural Science, Gyeongsang National University, Jinju 52828, Republic of Korea

**Keywords:** Northeast asia, Greater khingan, *Populus koreana*, Quaternary, Glacial refugia

## Abstract

The genetic structure of temperate plants in the northern hemisphere was significantly influenced by the Quaternary climate oscillations. A species' biological characteristics and ecological niche are significant elements that can affect its phylogeographic history. We adopted the cold-tolerant, anemophilous and anemochorous tree, *Populus koreana*, as a model species to examine the impact of historical climate changes and biological characteristics on the evolutionary history of vegetation in Northeast Asia throughout the Quaternary period. The results showed that there is moderate genetic differentiation and a lack of phylogeographic structure among populations of *P. koreana* based on nuclear microsatellite and plastid markers. Demographic analyses and ecological niche modeling suggested that *P. koreana* is likely to have experienced a bottleneck around the last glacial maximum (LGM), followed by a rapid and continued range expansion coupled with a northward migration from the LGM to the mid-holocene (MH), present, and 2050. Notably, there were several separate refugia present throughout the range of *P. koreana* in Northeast Asia during the LGM. These include two widely recognized refugia located in the Changbai Mountains and the southern Korean Peninsula. We also unexpectedly found a previously unknown one in the northern Greater Khingan Mountains. Our study contributes to the understanding of the phylogeographic history of plant species in Northeast Asia, providing novel insights into the Greater Khingan Mountains as glacial refugia for a cold-tolerant tree species. These findings provide valuable insights into the Quaternary historical patterns of temperate forests in East Asia.

## Introduction

The distribution of genetic variation across populations within a species are primarily shaped by historical (e.g. demographic fluctuations due to changing climate) and contemporary factors (e.g. population connectivity and/or effective population size)^[1,2]^. The impact of climate oscillations during the late Tertiary and Quaternary periods had a notable effect on the geographic distribution and genetic structure of species in East Asia, despite a considerable area of the region remaining ice-free^[3−5]^. This resulted in the contraction of populations and their isolation in multiple glacial refugia, followed by the expansion during more favorable periods^[6,7]^. Additionally, the rise and fall of sea levels had an impact on the connectivity between regions influencing migration patterns and differentiation of populations, especially for peninsulas and islands^[8]^. These factors may generate repeated genetic bottlenecks leading to genetic differentiation between populations^[4,9]^.

Phylogeographic studies have shown that some coniferous species such as *Pinus tabuliformis* Carriere and *Picea asperata* Mast*.*^[6,10−12]^ had several glacial refugia within mainland China throughout the Quaternary glacial cycles. There is a growing body of evidence indicating the presence of microrefugia, also known as cryptic refugia, throughout Europe and North America, particularly at higher latitudes^[13−16]^. In addition to glacial refugia, postglacial/interglacial range expansion and admixture between populations that survived in different glacial refugia also shaped the genetic diversity patterns^[5]^. This is more pronounced for species with strong dispersal ability, such as wind-dispersed (anemophilous and anemochorous) species^[17]^. There is accumulating evidence that even in sympatry those species may experience different evolutionary trajectories and phylogeographic histories^[18,19]^. For example, high-latitude populations of *Picea abies* and *Alnus incana* in Northern Europe were shown to have different origins, with the former originating from Fennoscandian populations and the latter from Central European ones^[20]^.

In East Asia, many phylogeographic studies concentrated on glacial refugia in West China but very few investigated Northeast China and the Korean Peninsula^[15,21,22]^. Although there are no large geographic barriers in Northeast China and the Korean Peninsula, the distribution patterns for genetic diversity in various species are complex and diverse^[7,23]^. Combining evidence from genetic data and ecological niche models (ENMs), some studies showed that plants in coniferous and broad-leaved mixed forests had a single refugium in the Changbai Mountains (also titled the Baekdu Mountains on the Korean side)^[6,15,24]^. In addition, other studies indicated that there may have been other refugia in the Korean Peninsula (also known as 'Baekdudaegan', the main Korean mountain range)^[25,26]^, and the Russian Far East^[7]^. To investigate the existence of smaller microrefugia within species, a larger number of genetic data coupled with adequate sampling throughout the distribution range is needed^[21]^.

**Figure 1:**
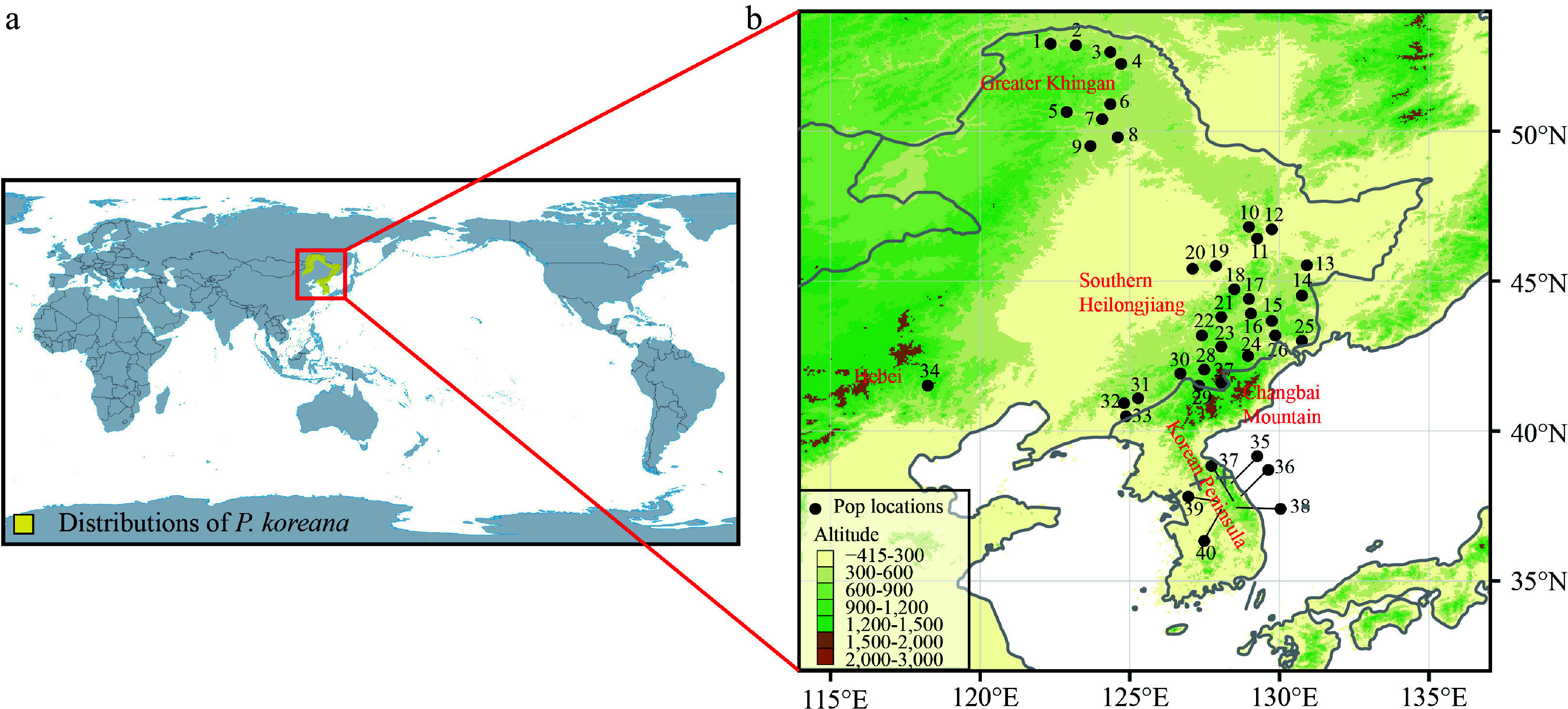
(a) The main distribution range of *Populus koreana*. (b) Map of the 40 sampled *P. koreana* populations. Supplemental Table S1 contains the code and coordinates of each population.

*Populus koreana* Rehd. (Salicaceae), a tree species characterized by anemophily and anemochory, is found in the northeastern region of China, the southern Korean Peninsula, and the southernmost area of the Russian Far East (Fig. 1a). In China, the primary distribution occurs in Heilongjiang and Inner Mongolia Provinces (the Greater Khingan Mountains), Jilin Province (the Changbai Mountains), and the eastern part of Liaoning province. In the distribution range of *P. koreana*, the Greater Khingan Mountains are quite isolated from other populations and located farther north (Fig. 1b). Given the common distribution of this species there, we cannot rule out the possibility that it existed as glacial refugia throughout the LGM. Alternatively, the existing geographic range of *P. koreana* within the specified region can be explained by the process of postglacial recolonization from southern populations. This recolonization was made possible by the species' significant capacity for dispersal. Hence, *P. koreana* serves as a suitable model species for investigating the hypothesis that the Greater Khingan Mountains have functioned as refugia for coniferous and broad-leaved mixed forests in Northeast Asia.

In order to examine the phylogeographic pattern and demographic history of *P. koreana*, we used nuclear microsatellites (nSSRs) and chloroplast DNA (cpDNA) sequences together with ENMs. Specifically, we aimed to answer: (1) What is the population genetic structure for *P. koreana*? (2) Is there genetic and ecological evidence for multiple refugia of *P. koreana* during the late Quaternary, particularly the Greater Khingan? (3) How did the species' distribution range change due to the Quaternary climate oscillations and how might it be affected in the future?

## Materials and methods

### Sample collection and DNA extraction

To determine the present distribution range of *P. koreana*, an investigation was conducted using the Chinese Virtual Herbarium of (https://www.cvh.ac.cn), the Plant Photo Bank of China (https://www.ppbc.iplant.cn), and the Global Biodiversity Information Facility (https://www.gbif.org). These sources were consulted to retrieve relevant records of *P. koreana*. We collected leaf samples of 424 adults from 40 natural populations in Northeast China (34 populations: The Greater Khingan [Pop1 to Pop 9]; southern Heilongjiang province [Pop10 to Pop20]; the Changbai Mountains [Pop21 to Pop33]; Hebei province [Pop34]) and the southern Korean Peninsula (six populations: Pop35 to Pop40) (Fig. 1b, Supplemental Table S1). In order to avoid the sampling of clones, a minimum distance of 30 m was upheld between adjacent individuals. The leaves were collected and thereafter stored in silica gel at ambient temperature until the extraction of DNA was performed. Approximately 20 mg of leaf tissue was utilized to extract genomic DNA using the CTAB method^[27]^.

### Microsatellite screening and chloroplast DNA sequencing

A total of 11 nuclear microsatellite loci were utilized to survey the genetic variation of *P. koreana* populations (Supplemental Table S2). The polymerase chain reaction (PCR) was performed for all nuclear microsatellite's loci in a 25 μL solution. The solution consisted of 50−100 ng of diluted plant DNA, 0.5 mM of each deoxyribonucleotide triphosphate (dNTP), 0.5 μL of each primer, 2.5 μL of 10 × Taq buffer, and 0.5 units of Taq polymerase (Vazyme Biotech, Nanjing, China). The experimental procedures were conducted utilizing a Master Cycler Pro (Eppendorf, Hamburg, Germany) thermal cycler. The thermal cycling settings consisted of an initial denaturation step at 95 °C for 5 min, followed by 35 cycles of denaturation at 95 °C for 45 s, annealing at 55 °C for 30 s, and extension at 72 °C for 45 s. A final extension step was performed at 72 °C for 10 min. Following that, the PCR results were evaluated using a 1% agarose gel and subsequently forwarded to Tsingke Biotech (Beijing, China) for fragment size polymorphisms analysis.

The standard primers for the three cpDNA regions were used for amplification and sequencing (Supplemental Table S3). PCR for all cpDNA fragments followed the same procedure as for SSR amplification. The DNA sequences of the three regions were combined and aligned using the ClustalW technique included in the CLUSTAL X software^[28]^. All DNA sequences have been submitted to the National Center for Biotechnology Information (NCBI) with GenBank accession numbers: OP356757–OP357897.

### Microsatellite analysis

The determination of the frequency of null alleles (*F*_null_) for each locus was conducted using CERVUS v.3.0^[29]^. The estimation of genetic diversity indices was conducted using GenAlEx v.6.501^[30]^. These indices included the number of alleles (*N*_a_), the effective number of alleles (*N*_e_), expected heterozygosity (*H*_e_), observed heterozygosity (*H*_o_), inbreeding coefficient at the population level (*F*_IS_), the proportion of differentiation among populations (*F*_ST_), and gene flow (*N*_m_). Gene flow was determined using the formula *N*_m_ ≈ [(1/*F*_ST_)-1]/4, where *N* represents the effective population size and *m* represents the proportion of migration per generation^[31]^. The genetic diversity of each population was calculated by averaging the values across all 11 nSSR loci.

The calculation of allelic richness (*A*_r_) and rarefied private allelic richness (*P*_ar_) of each population was performed using HP-Rare 1.1^[32]^. In order to visually represent the spatial distribution of genetic diversity, we employed the inverse distance weighting (IDW) technique in ArcGIS v.10.2.2 software. This allowed us to generate a spatial interpolation for *H*_e_ and *A*_r_ values. In order to assess the potential association between within-population genetic diversity and geographic distance, we computed Pearson's correlation coefficients (*r*) between the genetic diversity measures *H*_e_ and *A*_r_, and the latitude for each population. This analysis was performed using the ade4 program^[33]^. In order to investigate the correlation between genetic and geographic distance among populations, we utilized a Mantel test^[34]^ to test the presence of Isolation by distance (IBD). Significance was determined using 10,000 permutations.

The investigation of population genetic structure was conducted using the software program STRUCTURE v.2.3.4^[35]^. The program was executed a total of 20 times for each *K*-value, ranging from 1 to 10, across all populations. The execution involved a burn-in period of 500,000 iterations, followed by 5,000,000 Markov Chain Monte Carlo replicates after the burn-in phase. The program utilized the 'admixture' and 'correlated allele frequencies' parameters. The ideal value of *K* was ascertained by employing likelihood plots and the Δ*K* method^[36]^, with the assistance of the web-based software STRUCTURE HARVESTER^[37]^. In order to visually represent the genetic linkages both within and across different geographic groupings, principal component analysis (PCoA) was conducted using the genetic distance matrix derived from the SSR profiles. The data was later grouped into geographic categories using the adegenet package, which was built in the R programming language^[38,39]^. Nei's genetic distance^[40]^ was computed using Arlequin v.3.5^[41]^ with 1,000 bootstraps. The genetic links among clusters, which indicate the most probable number of unique genetic clusters *K*, were visually represented using a neighbor-joining (NJ) tree. The construction of this tree was based on genetic distances using the MEGA7 software^[42]^.

The study employed the hierarchical analysis of molecular variance (AMOVA)^[43]^ to evaluate the distribution of genetic variation among populations. This analysis included the calculation of *Φ*_ST_, which represents the ratio of the variance component among populations to the total genetic variance in the sample. The assessment was conducted separately for four distinct regions to examine both the partition of genetic variation among populations and within populations. The analysis was conducted using Arlequin v.3.5.1 software^[41]^ for the nuclear single sequence repeat (nSSR) datasets. The AMOVA analysis was conducted in the entire sample, employing a hierarchical approach with three levels. These levels included analysis among regions, analysis among populations within regions, and analysis within populations. The statistical significance was assessed using 1,000 permutations.

### Chloroplast DNA marker analysis

In order to assess the variability in genetic diversity among populations, the software DnaSP v.5.10^[44,45]^ was employed to compute several metrics including the number of polymorphic sites (S), the number of haplotypes (H), nucleotide diversity (*P*_i_), and haplotype diversity (*H*_d_). Arlequin v.3.5.1^[41]^ and MEGA7^[42]^ were employed for the computation of *F*_ST_. The haplotype network using the median-joining method was computed using Network v.4.6^[46]^. The coefficients of differentiation *G*_ST_ and *N*_ST_ were computed using the PERMUT statistical method^[47]^ in order to examine the presence of phylogeographic structure within the dataset. The AMOVA analysis was conducted on the cpDNA datasets in the nSSR dataset using Arlequin v.3.5.1^[41]^ (significance test with 1,000 permutations).

### Demographic history

**Figure 2:**
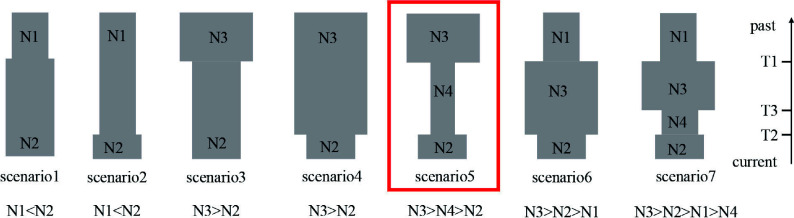
Using 11 nSSR loci and DIYABC2.0, a schematic representation of the seven demographic scenarios for *P. koreana* was examined, along with model parameters.

In order to investigate the demographic history of *P. koreana*, we utilized the nSSR dataset and employed the Approximate Bayesian Computation (ABC) method using DIYABC v.2.1.0 software^[48]^. A total of seven distinct scenarios about the demographic history of *P. koreana* were tested (Fig. 2). The posterior distribution of the coalescent parameters was determined from the subset using a local linear regression technique. The posterior probabilities of the seven scenarios were subsequently assessed by calculating the relative frequency of simulated datasets corresponding to each scenario within the 500 datasets that closely resembled the observed dataset. In addition, logistic regression was conducted to estimate the probability of each scenario. This estimation was based on the differences between observed and simulated summary statistics^[49]^. The evaluation of the reliability of our ultimate scenario was conducted by computing errors based on posterior and prior probabilities, as well as scenario-specific type I and type II mistakes. This analysis was performed utilizing supplementary pseudo-observed data sets constructed for each scenario. The prior values utilized for all of these factors are documented in Supplemental Table S4. Summary statistics were calculated for each scenario in order to determine the most probable scenario and estimate the posterior distribution of demographic characteristics.

Tajima's *D*^[50]^, Fu & Li's* D*^[51]^, and Fu & Li's *F*^[51]^ were estimated using cpDNA datasets and the program DnaSP v.5.0^[44,45]^. Significant negative values were taken as evidence for demographic expansion and positive values for a potential population bottleneck. Additionally, 1,000 bootstrap repeats of a mismatch distribution analysis in Arlequin were used to look for expansion. Sums of square deviations (SSD) and Harpending's raggedness index (H_Rag_) were used in the research to evaluate the relevance of two factors^[52]^. The methodology involved the linear fitting of observed and simulated curves.

### Ecological niche modeling

In order to investigate the potential distribution range shift during the Quaternary period, the distribution range of *P. koreana* at present (1970–2000), during the last interglacial period (LIG: ca. 130 thousand years before present, kya), during the LGM (ca. 21 kya), the Mid-Holocene (MH: ca. 6 kya), and the future (2050) with all 19 bioclimatic variables were modeled using ENMs in Maxent v.3.3.3 ^[53] ^(Supplemental Table S5). The localities that we used to predict distributions are the sampling sites in this study (Supplemental Table S1).

The climatic variables were acquired from Worldclim v. 2.0, which was accessed at the website http://www.worldclim.org/. The data was gathered at a resolution of 2.5 arc seconds. The pairwise correlation between variables was evaluated using ENM Tools v.1.4.4. Environmental variables that exhibited strong autocorrelation (Pearson's *r* > 0.75) were eliminated from further analysis. The final models incorporated five climatic variables, namely bio3, bio4, bio5, bio13, and bio19 (refer to Supplemental Table S6). The evaluation of the model's performance was conducted by utilizing the area under the receiver operating characteristic curve (AUC) and threshold-dependent binomial omission tests, which were generated using Maxent ^[53]^. The AUC varies between 0.5, which signifies that places are equally probable to be classified as presence or absence, and 1.0, which indicates a flawless classification of presence and absence. In general, an AUC value exceeding 0.7 signifies a satisfactory level of model robustness^[54]^.

## Results

### Nuclear microsatellite data

All microsatellite loci were used for subsequent analysis since there was little evidence of null alleles (Fnull of each nSSR locus was < 0.4; Supplemental Table S7)^[55]^. The mean number of alleles, denoted as *N*_a_, was found to be 4.51 (with a range of 2.82–6.00), while the average number of migrants each generation, denoted as *N*_m_, was determined to be 1.55 (with a range of 0.61–2.15). The mean observed heterozygosity (*H*_o_) and expected heterozygosity (*H*_e_) of the entire population (*n* = 424) were found to be *H*_o_ = 0.514 and *H*_e_ = 0.569, respectively (Supplemental Table S7). In addition, *F*_ST_ across all loci was *F*_ST_ = 0.150 (0.104–0.290).

The mean observed heterozygosity (*H*_o_) of the 40 populations at the population level was determined to be 0.514. Among these populations, Pop18 exhibited the greatest observed heterozygosity value of 0.627, while Pop38 had the lowest value of 0.400 (Supplemental Table S1). The observed levels of heterozygosity (*H*_e_) varied across populations, ranging from 0.417 in Pop3 to 0.647 in Pop15, with an average value of 0.569 (Supplemental Table S1). The average *F*_IS_ of the 40 populations of *P. koreana* was 0.103. All six populations from the southern Korean Peninsula showed higher than average *F*_IS_ values (Supplemental Table S1), suggestive of higher levels of inbreeding. In contrast, the inbreeding coefficients of populations distributed in Northeast China (Pop5, Pop13, Pop16, Pop18, Pop31, and Pop33) were 0 (Supplemental Table S1), suggesting probable random mating.

We demonstrated that the genetic diversity of *P. koreana* populations in Northeast China was often greater than that of the populations found on the southern Korean Peninsula (Supplemental Fig. S1) using the IDW method, with *H*_e_ and *A*_r_ as indicators. In Northeast China, populations on the Changbai Mountains and three populations from the Greater Khingan (Pop1, Pop8, and Pop9) had higher IDW values (Supplemental Fig. S1), although the genetic diversity of populations inhabiting the Changbai Mountains exhibited a usually higher level in comparison to the groups residing in the Greater Khingan Mountains. The Mantel test revealed a significant positive correlation between genetic distance and geographical distance (*R*^2^ = 0.129, *P* = 0.001) (Supplemental Fig. S2), suggesting the presence of isolation-by-distance. The results of the Pearson correlation test indicated that there was no significant correlation between *H*_e_ (*R*^2^ = –0.157, *P* = 0.335) and *A*_r_ (*R*^2^ = –0.125, *P* = 0.443) with latitude (Supplemental Fig. S3).

**Figure 3:**
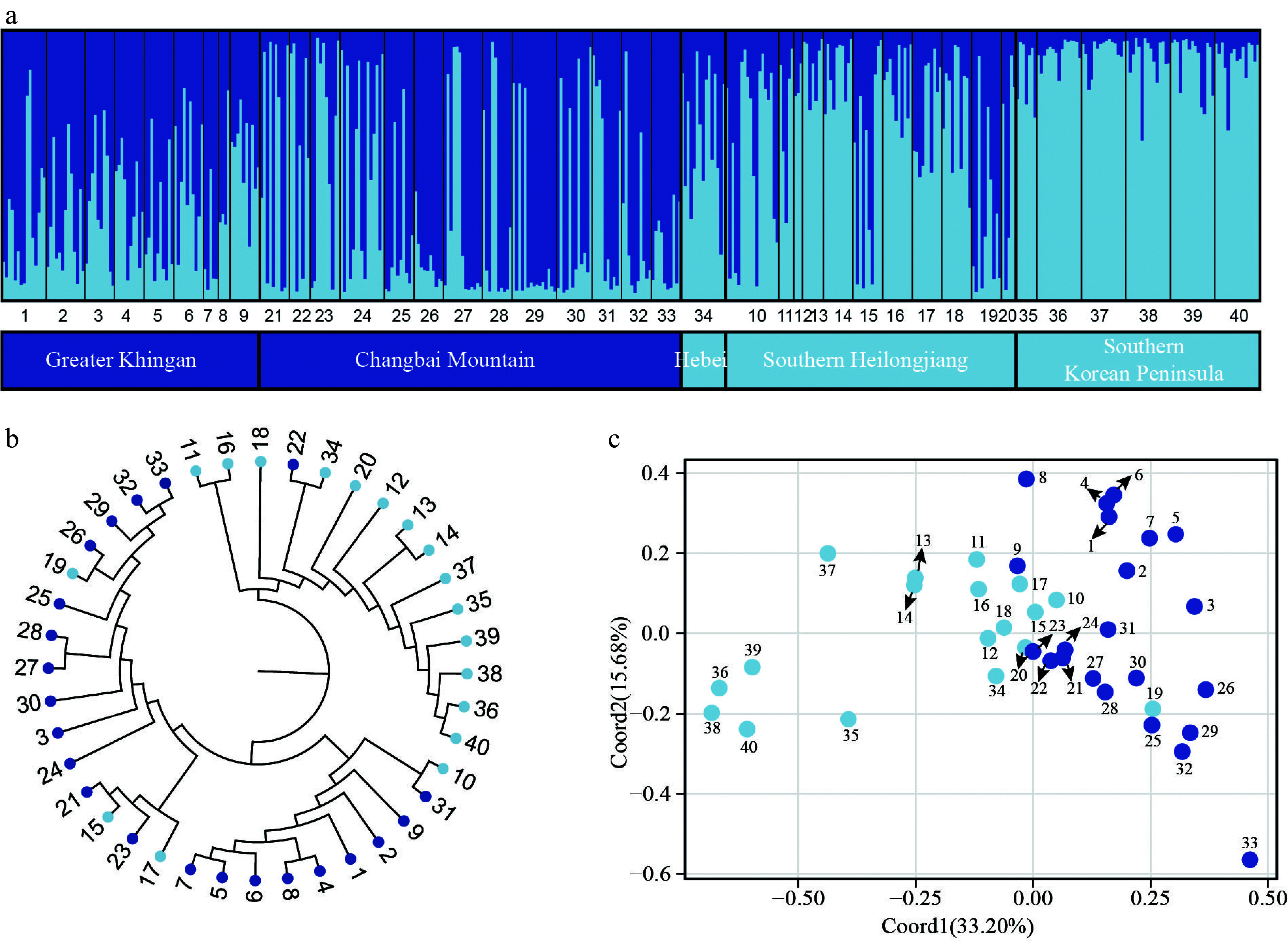
Population genetic structure of *P. koreana* based on 11 nSSRs (*n* = 424). (a) The histogram illustrates the outcomes of the STRUCTURE assignment test with a value of *K* = 2. The representation of each individual is denoted by a vertical bar, which signifies the cumulative assignment probabilities to the two groups. The utilization of black lines serves the purpose of demarcating distinct populations. (b) The construction of a phylogenetic tree representing all populations of *P. koreana* based on DA. The genetic clusters found by STRUCTURE analysis are demarcated by branch colors. Please refer to Supplemental Table S1 for the corresponding population codes. (c) Principal coordinate analysis (PCoA) was conducted on a dataset consisting of 40 populations. The results revealed that Coord1 accounted for 33.20% of the variation, while Coord2 explained 15.68% of the variance.

Based on the analysis of the nSSR data using the STRUCTURE method, it was determined that the most probable number of genetic clusters for *P. koreana* is *K* = 2 (Supplemental Fig. S4) with one group consisting of populations distributed in the Greater Khingan and the Changbai Mountains, and the other group including populations from southern Heilongjiang (the northern Changbai Mountains) and the southern Korean Peninsula. However, there were many individuals belonging to both genetic groups in most populations (Fig. 3a). When *K* = 3, the Greater Khingan and the southern Korean Peninsula populations could be mainly distinguished, with populations in southern Heilongjiang containing varying proportions of all three groups (but seemed to be more similar to those in the southern Korean Peninsula; Supplemental Fig. S5). The NJ tree, derived from genetic distances across all populations, exhibited two distinct clusters, a finding that aligns with the results obtained from the STRUCTURE analysis (Fig. 3b) as well as the observed geographical distribution patterns.

AMOVA, analyzed for four regions separately, revealed that populations from the Changbai Mountains (*Φ*_ST[nSSR]_ = 0.066) and Greater Khingan Mountains (*Φ*_ST[nSSR]_ = 0. 067) had slightly higher *Φ*_ST[nSSR]_ values than others regions (Supplemental Table S8). The AMOVA analysis revealed that approximately 26% of the genetic variation was distributed throughout populations (*Φ*_ST[nSSR]_ = 0.257, *P* < 0.001) for all individuals (Supplemental Table S8).

### Chloroplast DNA data

**Figure 4:**
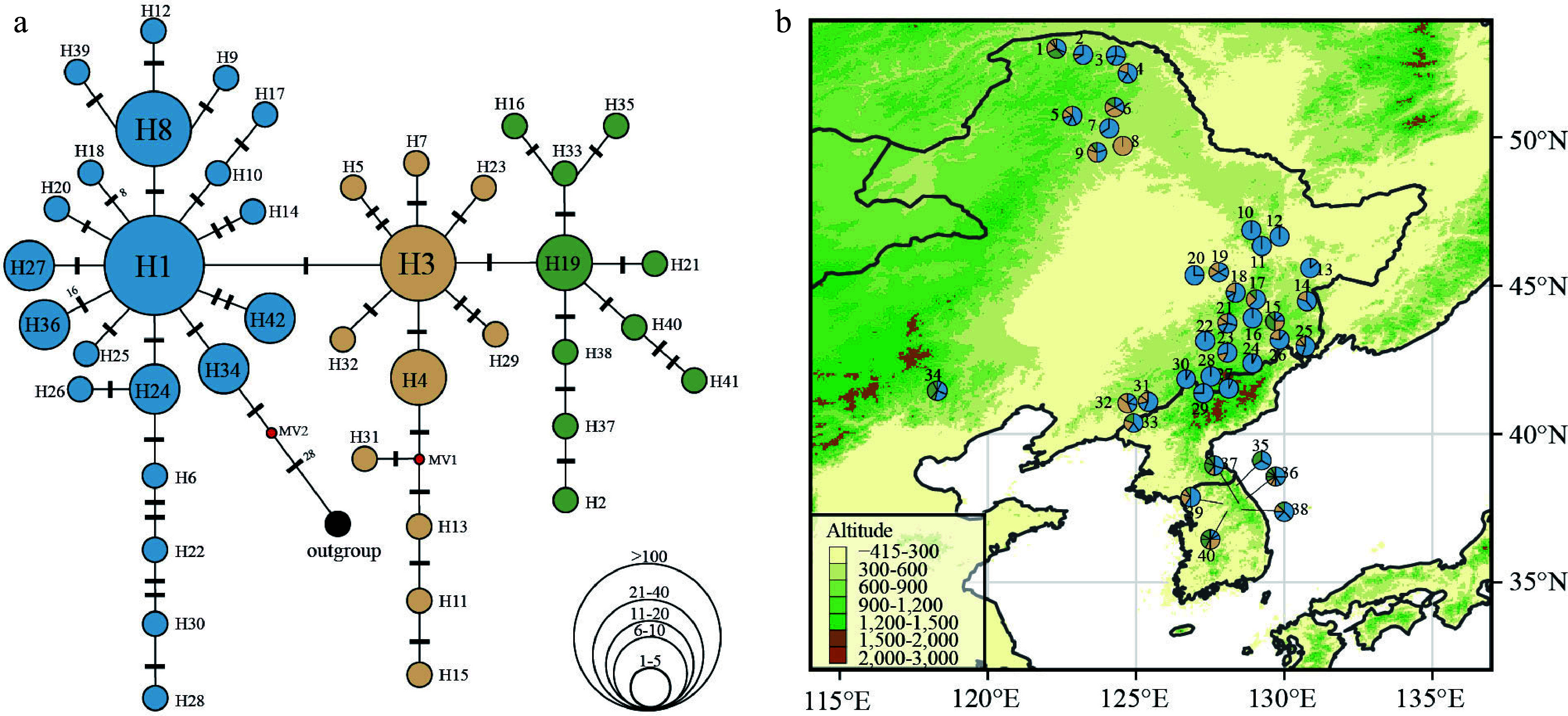
(a) Chloroplast haplotype network of 42 haplotypes discovered in 40 *P. koreana* populations (n = 398). The size of the circles represents the relative frequency of each haplotype, while the presence of red dots signifies the absence of certain haplotypes. Bars indicate the number of mutations between haplotypes. (b) Haplotype frequency distribution in 40 *P. koreana* populations, colors correspond to the haplotype colors in (a).

In total, 85 haplotypes were obtained from 398 individuals (40 populations). Discarding singleton haplotypes (i.e., haplotypes that were only present in one individual) we obtained 42 haplotypes for further analysis. The range of haplotypes (H) per population varied from one to nine (Supplemental Table S9). Among the 40 populations, 33 populations had more than two haplotypes, and seven populations harbored only one haplotype. The most frequent haplotype was haplotype 1 (H1), which is likely to be of ancestral origin due to its central position in the haplotype network (Fig. 4a) and occurred in 90% of the populations (Fig. 4b). Among the 40 groups examined, it was observed that 15 of them, accounting for 37.5% of the total, had private haplotypes. A total of nine distinct haplotypes were identified in the Greater Khingan region, four in the southern Korean Peninsula, and six in the Changbai Mountains. These findings indicate the presence of glacial refugia in these specific geographical locations during the LGM (Supplemental Table S9).

The haplotype diversity (*H*_d_) had a mean value of 0.658, whereas the average nucleotide diversity (*P*_i_) was estimated to be 0.00193 (Supplemental Table S9). Based on the datasets that do not include single mutations (referred to as 'parameter b'), the average gene diversity within populations (*H*_S_ = 0.624) was found to be lower than the overall gene diversity (*H*_T_ = 0.752). This resulted in a moderate level of *G*_ST_ (0.170) for *P. koreana* populations (Supplemental Table S10). Importantly, this value was not significantly different from *N*_ST_ (*N*_ST_ = 0.201, *P* > 0.05), suggesting a lacking of phylogeographic structure, which means that closely related haplotypes are not tend to occur in the same region. Analysis of the dataset including unique mutations ('parameter a') did not change this interpretation (Supplemental Table S10). AMOVA revealed that the degree of population differentiation (*Φ*_ST[cpDNA]_ = 0.228, *P* < 0.001) calculated based on cpDNA data was slightly lower than *Φ*_ST[nSSR]_ (0.257, *P* < 0.001) estimated from nSSR data (Supplemental Table S8).

### Demographic history

Based on the analysis of cpDNA data, it can be inferred that *P. koreana* underwent a range expansion (Supplemental Table S11). The examination of mismatch distribution, using datasets based on 'parameter b', revealed that the values of SSD (0.11) and H_Rag_ (0.15) were not statistically significant (*P* > 0.05) (Supplemental Fig. S6 & Table S11). This suggests that *P. koreana* underwent population expansion in its evolutionary history. In contrast, DIYABC suggests an ancient bottleneck and then a recent expansion. Logistic regression, direction estimate (Supplemental Fig. S7), and principal component analysis (PCoA) plots (Supplemental Fig. S8) strongly supported scenario 5 as the most likely scenario out of the seven models (Fig. 2; Supplemental Table S12). In this scenario, *P. koreana* experienced an ancient bottleneck of ca. 21.25 kya and went through a recent expansion of ca. 6.02 kya (Fig. 2; Supplemental Table S13). The aforementioned dates align with the geological occurrences of the LGM and the MH, correspondingly.

### Ecological niche modeling

**Figure 5:**
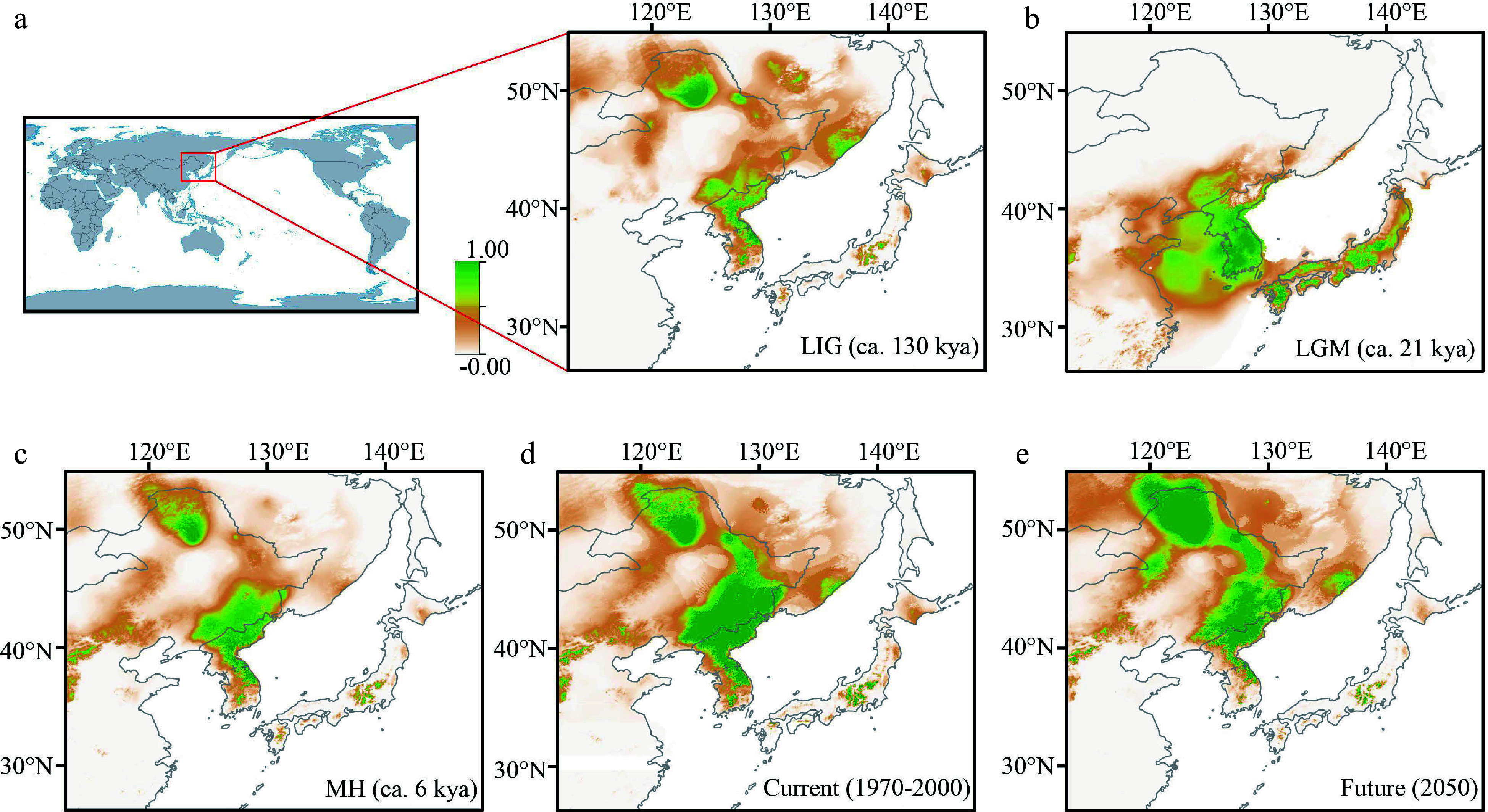
Ecological niche modeling outputs of *P. koreana* (a)−(e) under (a) LIG, (b) LGM, (c) MH, (d) present and (e) future conditions, respectively. Warmer colors indicate higher probabilities of occurrence, and orange and yellow indicate medium and low probabilities, respectively.

The mean AUC value for the present distribution was 0.941, suggesting that the model's predictions align well with the actual distribution of the species. During the previous interglacial period, known as the LIG approximately 130,000 years ago, the suitable environments for *P. koreana* were limited to the Khingan Mountains and the Baekdudaegan, a mountain range located on the Korean Peninsula, and adjacent regions (Figs 1a & 5a). During the LGM, the habitat that was suited for *P. koreana* experienced a significant contraction, resulting in the species being primarily confined to the southern Korean Peninsula and potentially extending to Japan (Fig. 5b). During the MH period, the habitat that was suitable for the species was significantly bigger in comparison to the LGM period. As a result, the species was able to extend its range to include the Changbai Mountains and the Greater Khingan region (Fig. 5c). From the MH to the present, there has been an increase in the distribution range of the species (Fig. 5d). According to the models, it is predicted that by 2050, the species will continue to expand northward, with minor contractions in the southern and central parts of its current distribution (specifically, the southern Korean Peninsula and the Changbai Mountains; as depicted in Fig. 5e). The findings suggest that *P. koreana* exhibited a characteristic pattern of contraction in response to glacial periods and expansion during interglacial/postglacial periods.

## Discussion

This study employed molecular phylogeography, demographical history analysis, and ENMs to examine the genetic diversity patterns, glacial refugia, and range dynamics of *P. koreana*, a deciduous tree species known for its cold tolerance. We discuss these issues first and test 'the Greater Khigan Mountains-refugia hypothesis' in the two sections below.

### Molecular markers reveal a clearer population genetic structure

The genetic diversity of *P. koreana*, as determined by nSSRs (*H*_e_ = 0.569) and chloroplast DNA (*H*_s_ = 0.624), was found to be similar to or slightly lower than the genetic diversity observed in *Populus* species and other endemic species in Northeast China (Supplemental Tables S1, S10). For example, *Populus davidiana* had values of *H*_e_ = 0.56 and *H*_s_ = 0.71^[19]^, and *Quercus mongolica* of *H*_e_ = 0.746 and *H*_s_ = 0.753^[7]^. The potential for gene flow between populations of *P. koreana* is likely to be high due to the wind dispersal of both pollen and seeds, as well as the relatively uniform topography between Northeast China and the Korean Peninsula in comparison to southwestern China. This might be consistent with the moderate level (compared to other trees examined by nSSRs: mean *G*_ST_ = 0.19)^[56]^ of genetic differentiation among populations (*Φ*_ST[nSSR]_ = 0.257; *Φ*_ST[cpDNA]_ = 0.228), and could explain the moderate levels of genetic diversity. It is widely acknowledged that a large proportion of trees exhibit moderate to high levels of genetic diversity^[57,58]^.

Typically, genetic differentiation (*F*_ST_, *G*_ST_, or its analogue *Φ*_ST_) of populations based on plastid markers is higher than based on nuclear markers^[59−61]^. The utilization of nuclear SSRs, which are markers inherited from both parents, might experience a higher level of gene flow in comparison to cpDNA, which is usually maternally inherited. This is expected as only seed flow should facilitates the dispersal of cpDNA whereas both seed and pollen flow will disperse nuclear markers, such as nSSRs. A phylogeographic study on *Populus lasiocarpa*, which occurs in the vicinity of the Sichuan Basin in southwestern China, reports a considerably higher *Φ*_ST[cpDNA]_ (0.763) than *F*_ST[nSSR]_ (0.276)^[62]^. A similar pattern was found in *P. davidiana* in Northeast China (*Φ*_ST[cpDNA]_ = 0.54; *Φ*_ST[nSSR]_ = 0.201)^[19]^. In our study region, comparable results were observed in other anemophilous tree species^[63−64]^. However, this trend is not supported by our results, suggesting a higher potential of wind-borne seed dispersal in the study region probably due to a relatively flat topography between the Greater Khingan and the rest of the regions. The microsatellite data of *P. koreana* showed gene flow (mean *N*_m_ = 1.552) was high as *N*_m_ > 1^[65]^. The genetic diversity patterns observed among populations are influenced by a range of factors, including fluctuations in effective population size, population structure, and the extent of gene flow. The findings of our study indicate that the increased cpDNA haplotype diversity observed in *P. koreana* can be attributed mostly to the occurrence of frequent gene flow among populations, and potentially even between sympatric poplar species^[66]^.

Based on the STRUCTURE analysis, it was determined that *K* = 2 (Fig. 3a) is the most probable number of genetic clusters. These clusters primarily differentiate populations from the southern Korean Peninsula and, to a lesser extent, populations from southern Heilongjiang. Other populations generally exhibit an admixture of the two genetic clusters. The results of the NJ tree and the PCoA were in alignment with the findings obtained from the STRUCTURE analysis. A Mantel test showed that geographically more distant populations were genetically less related but the relationship was weak (Supplemental Fig. S2). Although we sampled different mountain systems in Northeast China and the Korean Peninsula, no clear phylogeographic structure (*G*_ST_ = 0.170, *N*_ST_ = 0.201, *P* > 0.05) was detected based on cpDNA datasets. The cpDNA haplotype H1 was shown to be the most prevalent, occurring in 90% of the populations. Additionally, 25 out of the 42 cpDNA haplotypes were observed to be shared by two or more groups. CpDNA markers, which are maternally inherited, tend to 'survive' longer in introgressed populations than bi-parentally inherited nSSR markers which are more easily 'replaced' by higher levels of gene flow^[11,67,68]^.

### Multiple glacial refugia, including unexpected ones, and demographic history during Quaternary climate oscillations

There are two main hypotheses about the dynamics of distribution ranges in European and North American plant species in response to climate oscillations during the Quaternary period. One scenario that has been proposed is the '*tabula rasa'* hypothesis^[69]^, which suggests that plant species underwent extinction or range constriction at higher latitudes during glacial eras. However, these species managed to survive in lower latitude refugia and then recolonize higher latitudes during warmer interglacial/postglacial periods. The other scenario is that plant species survive '*in situ'* in micro-refugia or cryptic refugia (the 'nunatak' hypothesis)^[70−73]^ of higher latitudes during glacial times, and subsequently expand from these refugia during more favorable times. Usually, haplotype diversity (*H*_d_) and allelic richness (*A*_r_) will be higher in glacial refugia. Hence, if a species experienced a '*tabula rasa'* scenario (i.e. species survived in southern refugia and migrated northward after experiencing the glacial period), the genetic diversity within populations decreases significantly with increasing latitude^[15,21]^. In our research, it was observed that the ENMs is consistent with the '*tabula rasa'* scenario, suggesting that northern populations were eradicated during the LGM (Fig. 5b). However, it is important to note that there is no statistically significant negative correlation between genetic diversity and latitude (Supplemental Fig. S3). This is contrary to the expectation of 'high diversity in the south and low in the north'^[74]^.

Instead, our results support a scenario with multiple refugia during glacial periods. IDW analysis based on nSSR (Supplemental Fig. S1) suggested that the populations in the Changbai Mountains had higher genetic diversity than others and therefore may be considered a refugium during the LGM. Thus, it is probable that the Changbai Mountains have significantly influenced the genetic diversity and genetic structure of *P. koreana* populations. Meanwhile, Pop1, Pop2, Pop8, and Pop9 located in the Greater Khingan, Pop34 located in Hebei, and Pop35 located in the southern Korean Peninsula all had higher than average genetic diversity, which might be suggestive of survival in refugia or microrefugia.

The distribution pattern of cpDNA haplotypes also suggested *P. koreana* had multiple glacial refugia. The regions that were inferred as refugia had high haplotype diversity and several private haplotypes compared to the rest^[75]^. For example, populations where three or more haplotypes were found in the Greater Khingan (Pop1 to Pop6, Pop9), southern Heilongjiang (the northern Changbai Mountains; Pop14, Pop15, Pop17 to Pop19, and Pop21), Hebei (Pop34), the Changbai Mountains (Pop23, Pop25, Pop26, and Pop31 to Pop33) and the southern Korean Peninsula (Pop35 to Pop40; Supplemental Table S9). In addition, private haplotypes were found in several populations but still cover each of the above five regions (Supplemental Table S9). Two or more private haplotypes were found in the Greater Khingan (Pop1, Pop4), the Changbai Mountains (Pop32), and the southern Korean Peninsula (Pop36; Supplemental Table S9). If high haplotype diversity and private haplotypes are indicative of glacial refugia, then there may have been glacial refugia in each of the Greater Khingan, southern Heilongjiang (the southern Changbai Mountains), Hebei (the Yanshan Mountains), the Changbai Mountains (eastern Liaoning and eastern Jilin) and the southern Korean Peninsula. Among these, glacial refugia around the Changbai Mountains, Hebei, and the southern Korean Peninsula were reported before^[6,7,76−78]^. The findings from ENMs (Fig. 5) provide evidence in favor of the concept that the southern Korean Peninsula, specifically the Baekdudaegan region, acted as a glacial refugium for boreal and temperate plant species, including *P. koreana*, during the LGM (Fig. 5)^[25,26]^. Our study presents evidence that supports a previous untested glacial refugia in the Greater Khingan area for trees, despite prior findings indicating greater species diversity in this mountain range in comparison to neighboring locations^[79,80]^.

The ENMs of *P. koreana* during the LGM support glacial refugia in Hebei, the Changbai Mountains, and the southern Korean Peninsula, but not for the Greater Khingan and southern Heilongjiang. The southern region of Heilongjiang is in close proximity to the outer limits of its probable distribution range. In contrast, the Greater Khingan region is situated far farther north. This disparity implies that populations of *P. koreana* in the latter area may have endured and persisted in microrefugia during the LGM. However, *P. koreana* experienced a clear stepwise northward migration to southern Heilongjiang and further the Greater Khingan from the LGM (Fig. 5b) to the MH (Fig. 5c) and the present (Fig. 5d). The potential distribution in the Greater Khingan is expected to triple in size compared to the current distribution in the coming decades (2050, Fig. 5e). Meanwhile, the potential distribution of the southeastern range is predicted to contract in the coming decades (2050, Fig. 5e), which is consistent with a recent landscape genomic survey^[81]^. This phenomenon could potentially be associated with forthcoming alterations in environmental variables, including shifts in precipitation patterns and temperatures, and the burgeoning field of landscape genomics will offer a distinct line of evidence for forecasting future changes of forest distribution^[82]^.

Demographic inferences based on genetic data are consistent with ENMs. DIYABC analysis showed that *P. koreana* populations experienced a bottleneck of ca. 21.25 kya and a recent expansion of ca. 6.02 kya, which is close to the approximate time of the LGM of the Quaternary and the Holocene, respectively. The analysis based on the cpDNA data set indicates that populations of *P. koreana* may have undergone an expansion subsequent to a bottleneck event, as shown by the neutrality test. Despite the lower latitude of Northeast China and the Korean Peninsula compared to northern Europe and northern North America, which are known for their significant glaciation events, the demographic history of *P. koreana* exhibits similar patterns to plants that were greatly impacted by the climate changes during the Quaternary period.

### Limitations of this study

The taxonomic treatment of *P. koreana* in this study adhered to the guidelines outlined in the *Flora of China*^[83]^. However, the latest phylogenomic evidence suggests that the relationships among *P. koreana*, *P. suaveolens* Fisch. and *P. ussuriensis* Kom. are complex^[84]^, and the Plants of the World Online (POWO) database (and references therein) treated *P. koreana* as a synonym of *P. suaveolens*^[85]^. However, according to the *Flora of China*, there are obvious morphological differences between *P. koreana* and *P. suaveolens*^[83]^. The issue at hand pertains to a protracted discourse within the field of *Populus* taxonomy, including two distinct groups known as 'splitters' and 'lumpers'^[84]^. In this context, we argued that an inclusive examination, incorporating many operational criteria, offers a viable approach for the delimitation of poplars^[86]^. To better our understanding of species delimitation as well as the evolutionary history of *P. koreana*, *P. suaveolens*, and *P. ussuriensis*, we suggest that future studies may collect multiple lines of evidence, including but not limited to population genomic, morphological, ecological data, and populations across the whole distribution range of these species that cover China, Korea, Japan, Mongolia, and Russia, should be sampled.

## Conclusions

Both nSSRs and cpDNA analyses revealed that *P. koreana* exhibited a moderate proportion of genetic variation within populations and a moderate degree of genetic divergence between populations. The STRUCTURE analysis of nSSRs revealed two genetic lineages but cpDNA data did not indicate any obvious phylogeographic structure. This incongruent phylogeographic pattern between nSSR and cpDNA markers is likely to be associated with their differential levels of gene flow. Since the movement of genes between populations is a two-step sequential process *via* pollen and then by seeds, slightly lower cpDNA-based estimates (compared to nSSR-based ones) of among-population differentiation are suggestive of extensive seed flow across the landscape. Approximate Bayesian computation analyses and the neutrality test indicate that *P. koreana* experienced an ancient bottleneck and a recent expansion. ENMs also suggest rapid and continued range expansion after the LGM towards the north and northeast. The findings of our study provide evidence that three distinct refugia were preserved throughout the geographical distribution of *P. koreana* in Northeast Asia during the LGM. These refugia include the Changbai Mountains and the southern Korean Peninsula. Surprisingly, we also identify an hitherto unknown refugium in the Greater Khingan region. Therefore, our research on the phylogeography of a tree species with resilience to low temperatures, sheds new light on the Quaternary history of temperate forest in the East Asia.

## Author contributions

The authors confirm contribution to the paper as follows: study conception and design: Mao K, Wang Jing; data collection: Zhang H, Fan X; analysis and interpretation of results: Wang Ji, Wang S; draft manuscript preparation: Wang Ji, Mao K, Wang Jing, Ruhsam M, Chung JM, Chung MY, Chung MG. All authors reviewed the results and approved the final version of the manuscript.

## Data availability

Plastid DNA sequencing data have been deposited in the National Center for Biotechnology Information (NCBI) with GenBank accession numbers: OP356757–OP357897.
